# Natural Polysaccharide Carriers in Brain Delivery: Challenge and Perspective

**DOI:** 10.3390/pharmaceutics12121183

**Published:** 2020-12-06

**Authors:** Manuela Curcio, Giuseppe Cirillo, Jourdin R. C. Rouaen, Federica Saletta, Fiore Pasquale Nicoletta, Orazio Vittorio, Francesca Iemma

**Affiliations:** 1 Department of Pharmacy, Health and Nutritional Sciences, University of Calabria, 87036 Rende (CS), Italy; manuela.curcio@unical.it (M.C.); fiore.nicoletta@unical.it (F.P.N.); francesca.iemma@unical.it (F.I.); 2 Lowy Cancer Research Centre, Children’s Cancer Institute, UNSW Sydney, Sydney 2031, NSW, Australia; JRouaen@ccia.org.au (J.R.C.R.); FSaletta@ccia.org.au (F.S.); 3 School of Women’s and Children’s Health, Faculty of Medicine, UNSW Sydney, Sydney 2052, NSW, Australia; 4 ARC Centre of Excellence for Convergent BioNano Science and Technology, Australian Centre for NanoMedicine, UNSW Sydney, Sydney 2052, NSW, Australia

**Keywords:** polysaccharides, brain diseases, drug delivery, nanocarriers

## Abstract

Targeted drug delivery systems represent valuable tools to enhance the accumulation of therapeutics in the brain. Here, the presence of the blood brain barrier strongly hinders the passage of foreign substances, often limiting the effectiveness of pharmacological therapies. Among the plethora of materials used for the development of these systems, natural polysaccharides are attracting growing interest because of their biocompatibility, muco-adhesion, and chemical versatility which allow a wide range of carriers with tailored physico-chemical features to be synthetized. This review describes the state of the art in the field of targeted carriers based on natural polysaccharides over the last five years, focusing on the main targeting strategies, namely passive and active transport, stimuli-responsive materials and the administration route. In addition, in the last section, the efficacy of the reviewed carriers in each specific brain diseases is summarized and commented on in terms of enhancement of either blood brain barrier (BBB) permeation ability or drug bioavailability in the brain.

## 1. Introduction

The treatment of neurological pathologies often suffers from serious drawbacks related to the difficulty of delivering drugs across the blood brain barrier (BBB) to the site of interest at an effective concentration [1]. The BBB is a complex system composed of endothelial cells tight junctions, pericytes, and astrocytes which, together with the efflux proteins on the BBB surface, are considered the “security wall” of the central nervous system, regulating the entry of foreign substances in the brain [2]. Lipophilic xenobiotics can passively diffuse from the blood to the brain by crossing both the apical and basal membranes of endothelial cells [3]. On the other hand, hydrophilic compounds necessary for the neuronal functionality (e.g., glucose, α-amino acids, and vitamins) can be up-taken within the brain via specific cell membrane transporters [4]. Finally, high molecular weight molecules (e.g., peptide and proteins), which are too large for cell membrane transporters, can cross the endothelium to a limited degree through a vesicular route, either by specific receptor-mediated or adsorptive-mediated transcytosis [5].

Such a complex barrier, although being effective in protecting the brain from toxic molecules, hinders the entry of a large proportion of pharmacologically active molecules suitable for the treatment of many brain disease and disorders [6]. Thus, a significant challenge consists in designing therapeutic approaches to increase the number of drugs capable to overcome the BBB and/or improve their concentration in the brain. Biodegradable nanocarriers have been proposed as adequate solution to meet these needs by virtue of their small size and the ability to easily penetrate into cells, thus efficiently delivering drugs in the central nervous system [7]. Among others, natural polysaccharides (PS) were widely explored as base materials of drug delivery systems because of their biocompatibility, biodegradability, and muco-adhesion properties, with many of them being tested as neuroprotective and therapeutic agents in many cerebral pathological conditions [8], such as traumatic brain injury (TBI) [9], ischemia [10], neurodegenerative diseases [11,12], and glioma [13,14]. From an application point of view, despite some undeniable limitations such as the significant natural variability (e.g., the large range of molecular weights and the variation between batches), challenging synthesis and poor mechanical properties [15,16], PS shows favorable characteristics for the preparation of drug delivery systems. The presence of functional groups (hydroxyl, carboxyl, amino) allows tailored chemical derivatization, whereas the ability to interact with biological tissues via noncovalent bonding (e.g., electrostatic interactions) promotes the biorecognition of specific receptors on cell membranes. Furthermore, polysaccharide-containing carriers (PSC) have demonstrated enormous potential for drug delivery to the brain, with several mechanisms suggested for crossing the BBB [17,18].

**Figure 1:**
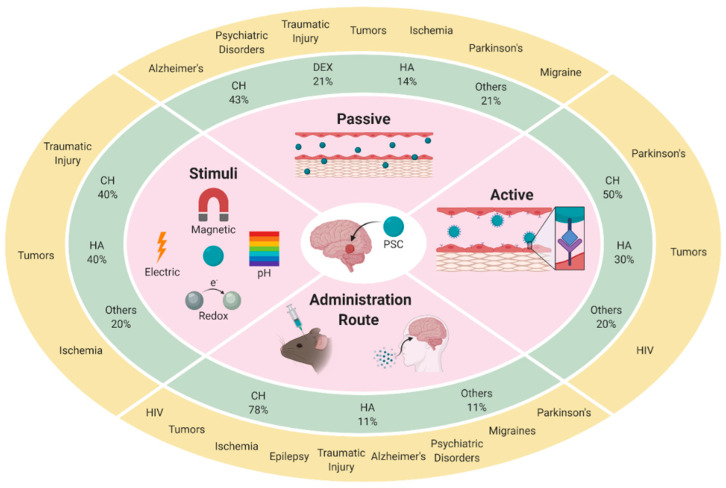
Overview of the main strategies (inner circle) for the vectorization of therapeutic agents to the brain. For each strategy, we report the frequency of use (%) of the different polysaccharide materials (middle circle) in the development of the polysaccharide-containing carriers (PSC) drug delivery systems and their application in the treatment of brain diseases (outer circle). CH: chitosan; DEX: dextran; HA: hyaluronic acid.

Different designs have been developed by using either the polysaccharide as unique component of nanosized carriers or combined with other organic or inorganic components in hybrid or core-shell systems. This review aims to give an overview about the PSC used in the treatment of the neurological diseases and disorders in the last five years, focusing on the main strategies to cross or bypass the BBB. In particular, we describe the carriers discussing the ability to target the BBB, the stimuli-responsivity, and the targeting efficacy modifying the administration route (Figure 1).

## 2. Targeting the Blood Brain Barrier

**Figure 2:**
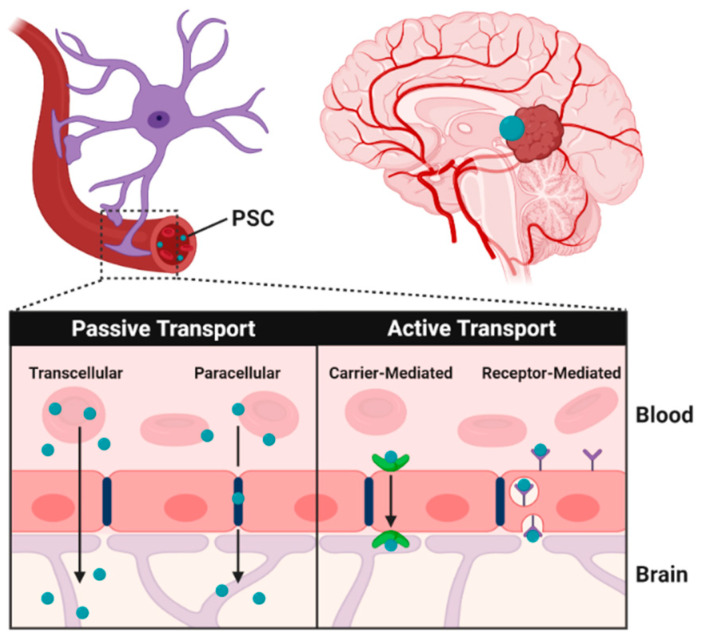
Passive and active transport of polysaccharide carriers across the blood brain barrier (BBB).

Similarly to other delivery systems, PSC can penetrate the BBB by two main mechanisms, namely the passive and active transport routes [19] (Figure 2).

The passive transport, an energy-independent process, involves the transfer of nanoparticles from the blood to the brain under a concentration gradient, whereas the active transport routes, involving the hydrolysis of adenosine triphosphate (ATP), include receptor- and carrier-mediated transport [20].

In Table 1, PSCs utilizing passive and active targeting or the treatment of brain diseases are reported, highlighting their efficacy in vitro and in vivo. For in vivo studies, the methodology employed for the evaluation of the carrier performance (e.g., improved animal behavior or analytical assessment of biochemical parameters) were specified.

### 2.1. Passive Targeting

Chitosan (CH) and CH derivatives were thoroughly investigated as base materials for the preparation of nanoparticles for brain delivery [48], with most of them formulated as passive-targeted carriers due to the ability to open endothelial cell tight junctions [49]. This phenomenon can be attributed to the high affinity of the negatively-charged BBB membrane for positively-charged compounds, which trigger cell internalization via adsorptive transcytosis [50]. In a recent work, memantine (MEM), a drug used in the treatment of Alzheimer’s disease, was loaded in CH nanoparticles obtained by γ-radiation and orally administered in a rat model. The formulation containing a drug-to-polymer 1:1 ratio was the most effective in transporting MEM into the brain, as evidenced by improved behavior performance and histopathological examination [21].

In another approach, the affinity, and thus the permeation across the BBB, was enhanced by the functionalization of the PSC with hydrophobic moieties. Lipidized CH [22] and dextran (DEX) [23,24,25], prepared by functionalization with alkylglycerols and formulated as nanoparticles, showed an increased drug permeability in both in vitro and in vivo experiments. Following the same approach, butylglyceryl derivatives of guar gum (GG) and pullulan (PL) were employed to develop nanocarriers loaded with a range of active agents (doxorubicin (DOX), rhodamine B (RhodB), angiotensin II (AngII)), and their performance as brain delivery systems was compared to that of the corresponding butylglyceryl CH nanoparticles. In vitro experiments showed that PL-based materials were characterized by improved cytocompatibility, hemocompatibility, and permeability [26]. CH, and the CH lipophilic derivative *N*-palmitoyl-*N*-monomethyl-*N*,*N*-dimethyl-*N*,*N*,*N*-trimethyl-6-*O*-glycolchitosan (PGCH), were also employed as coating materials to enhance the cell penetration of preformed nanoparticles. Hyaluronic acid (HA) nanoparticles were coated with CH and glycerol tripalmitin (GT), a biocompatible triglyceride present in human body fat, in order to increase the nanoparticle positive charge density and the system affinity for the BBB, respectively. The nanocarrier was loaded with oxygen-binding neuroglobin protein (NGB), a neuroprotective molecule against stroke and ischemia, and tested in vivo demonstrating an enhanced BBB crossing ability, as well as efficient penetration of damaged nerve cells 2 h post-injection) [27]. The amphiphilic PGCH was employed as a coating agent of peptide nanofibers bearing neuropeptides, showing that the PGCH coating enables the system to escape liver uptake, enhancing the plasma half-life and the brain delivery [28].

Other interesting examples of polysaccharide materials exploiting the passive targeting approach include alternative therapies for Parkinson’s disease [29,30] and glioblastoma (GBM) [31,32]. For instance, an injectable semi-interpenetrating hydrogel composed of HA and Collagen was used for delivery of Tat-Hsp70, a neuroprotective complex with high therapeutic potential in Parkinson’s disease [29]. Magnetic resonance imaging revealed that such composite can freely reach the brain and persist for at least 96 h, whereas in vivo experiments, using a model of dopaminergic degeneration, supported the role of hydrogel in conveying the neuroprotective effect of Tat-Hsp70 by both improving animal behavioral and dopaminergic neuronal integrity.

The encapsulation in maltodextrin (MDX) nanoparticles of tyrosine hydroxylase (TH), a brain enzyme catalyzing the synthesis of catecholamine neurotransmitters, including dopamine, DA, enhanced the enzyme stability either during nanoparticles storage or after internalization in neural cells, as demonstrated by the increased intracellular l-dopa synthesis following the nanoparticles uptake [30].

**Figure 3:**
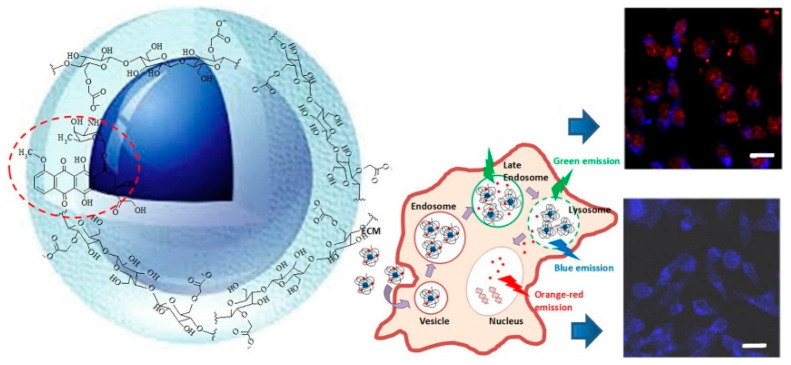
Representation of multifunctional nanovehicles for GMB theranostics based on a supramolecular colloid complex ZnS@CMC combined with DOX. Reproduced with permission from [31]. Elsevier (2019).

Multifunctional nanovehicles for theranostic applications in GBM treatment were obtained by coupling Carboxymethyl cellulose (CMC) or CMC conjugates with photoluminescent quantum dots based on ZnS and Ag-In-S (AIS). For example, the supramolecular colloid complex ZnS@CMC, combined with the anticancer drug Doxorubicin (DOX), was characterized by high biocompatibility and efficient internalization by brain cancer cells (Figure 3) [31].

Furthermore, vesicle-like nanoparticles, acting as “OFF–ON” intracellular nanosensors, were developed by employing CMC-peptide conjugates as surface capping ligands of ternary AIS quantum dots. The fluorescent nanoprobes were successfully used for targeted bioimaging, and intracellular tracking of GBM cells in vitro [32].

### 2.2. Active Targeting

Although promising, the passive targeting approach limited by low specificity of the interaction with the site of interest. Active transport mechanisms offer the opportunity to significantly enhance nanoparticles BBB penetration efficiency. This approach is based on carrier functionalization with specific ligands of receptors and/or transporters, involved in the brain metabolic activity and overexpressed on BBB endothelial cells to facilitate macromolecules and nutrients reaching the brain parenchyma in adequate concentrations.

The surfactant Polysorbate 80 (Tween80) is often employed as coating agent for actively targeting the nanoparticles to the brain due to its ability to absorb plasma apolipoprotein E (Apo-E) [51]. This allows nanoparticles to be recognized as low density lipoproteins (LDL) by LDL receptors of brain endothelial cells and internalized through a receptor-mediated endocytosis route with high efficiency [52]. 

Tween80-coated CH nanoparticles were proposed as carriers of nystatin (NYS) [34] and ropinolone hydrochloride (ROP) [35] demonstrating a significant drug accumulation in the brain in both cases. Moreover, in vivo biodistribution studies of ROP-loaded vehicles showed higher accumulation in brain compared to other organs such as liver, spleen and kidney [35]. An interesting neurotoxicity study highlighted the importance of the administered dose of Tween80-modified CH nanoparticles employed in neurodegenerative diseases. The results indicated an accumulation of nanoparticles in the frontal cortex and cerebellum after systemic injection, a dose-dependent weight loss of rats seven days after injection, as well as a dose-dependent neuron apoptosis and a minor inflammation of the frontal cortex [33].

Integrin receptors and amino acid transporters were also exploited as target for functionalized PSC in brain cancer. In particular, to obtain synergistic targeting and enhanced internalization by cancer cells, modified CMC was conjugated to DOX by amide bonds and functionalized with both arginylglycylaspartic acid (RGD) and l-arginine (l-Arg), an integrin target receptor tripeptide and a cell-penetrating amino acid, respectively [36]. The obtained system can form nanoparticles with hydrodynamic size ranging from 30 to 90 nm depending on pH and composition. In vitro experiments on healthy and cancer cells showed that the nanoparticles selectively address their cytotoxic potential towards the cancer cells, as a consequence of the presence of both the targeting moiety and internalization enhancer in their structure.

Interestingly, the carriers based on HA were successfully tested as targeted systems to treat glioma, exploiting its affinity towards CD44 receptors, overexpressed in tumor cell membrane [53], and the hydrolytic activity of Hyaluronidase, widely distributed in the acidic tumor extracellular matrix [54]. Curcumin (CUR) loaded nanoparticles based on CH-HA [37] and CH-HA-PEG [38] were proposed as carriers for the treatment of brain tumors. Although the study needed further investigation on the effective ability to cross the BBB, HA-mediated endocytosis was demonstrated in C6 cells, together with good pharmacokinetic parameters in an in vivo model. 

HA-DOX nanoparticles, obtained via nano-emulsification, were encapsulated in phospholipid structures to form liposomes coated nanoparticles, called LPsNP. LPsNP showed strong anti-cancer efficacy due to the accumulation in glioma cells and the regulation of the tumor microenvironment, with depletion of tumor associated macrophages, inhibition of vasculogenic mimicry channels and elimination of cancer stem cells [39]. Using the same structure, HA-coated liposomes (LNP) were employed as DOX nanocarrier and the uptake pathway was investigated in different brain cells, including primary astrocytes, microglia, and GBM cells. LNP showed a significant targeting effect against GBM cells due to higher expression of CD44, with an increased efficacy of DOX related to the lysosomal evasion, while in healthy cells limited toxicity was observed due to the absence of CD44 active uptake mechanism [40].

In order to enhance the target ability, HA was functionalized with HRK-19, a peptide containing RGD and NGR (Asn–Gly–Arg) motifs able to bind αvβ3 and aminopeptidase-N (CD13) receptors overexpressed in glioma cells and/or angiogenic vessels [41]. The obtained multi-target system possessed strong penetrative ability into the core of three-dimensional tumor spheroids and enhanced tumor localization after systemic administration in U87 tumor-bearing mice. Furthermore, when loaded with the antitumor drug Docetaxel (DTX), a negligible systemic toxicity and enhanced therapeutic efficacy was recorded, with significantly improved survival rates in intracranial C6 glioma-bearing rats. 

Peptide-functionalization was also employed to synthetize targeted Heparin (HP) nanoparticles derivatized with SWL and cRGD, two peptides able to target αvβ3 integrin and EphA2 receptors, respectively, overexpressed in glioma [42]. The proposed nanoparticles exhibited excellent glioma-targeting ability and anti-cancer efficacy due to the simultaneous inhibition of cell proliferation, lining of endothelial blood vessels, and vasculogenic mimicry.

Another important BBB target for drug delivery system is represented by the large amino acid transporter 1 (LAT1) [55]. LAT1 is overexpressed on the surface of the BBB endothelial cells, and facilitates the transport of amino acids such as leucine, isoleucine, valine, phenylalanine, tyrosine, and tryptophan. Thus, the functionalization of the PSC with such amino acids represents a suitable approach to enhance penetration efficacy and confer targeting properties to the resulting nanoparticle systems.

CH-L-Valine based nanoparticles were obtained by chemical crosslinking and loaded with saxagliptin (SGT), a dipeptidyl peptidase-4 enzyme inhibitor used in Alzheimer’s disease therapy. Nanoparticles can efficiently cross the BBB and are characterized by high stability in plasma, as demonstrated by in vivo and pharmacokinetic studies [43]. In another work, HA-*N*-Acetyl-l-Methionine (HA-ADH-AcMet) conjugate was investigated through binding affinity studies as targeted delivery system, showing high stability and superior binding towards LAT1 receptor compared to HA, Met and AcMet alone [44].

**Figure 4:**
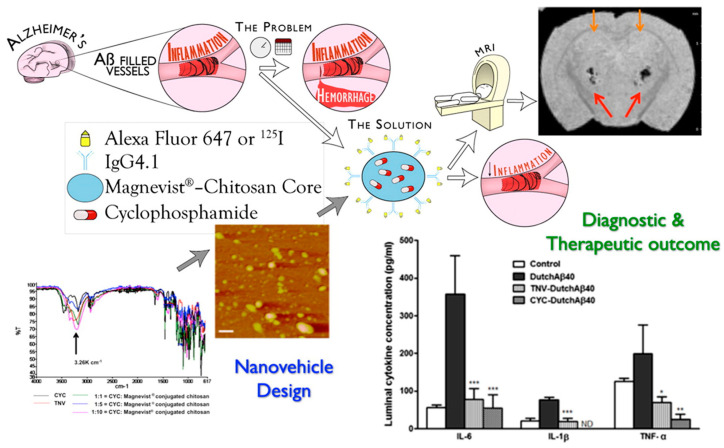
Effectiveness of theranostic nanovehicles based on CH-Magnevist^®^ in targeting cerebrovascular amyloid deposits. * *p* < 0.05, ** *p* < 0.01, *** *p* < 0.001. Reproduced with permission from [46]. Elsevier 2014.

Finally, targeted materials for a specific receptor can be achieved by coating or functionalizing the nanoparticles with antibodies, thus avoiding the competition between the targeted nanoparticles and free endogenous ligands. Hydrophilic theranostic nanovehicles composed of hydroxypropil-β-cyclodextrin and CH, bearing hydrophobic anti-inflammatory CUR and immunosuppressant (dexamethasone, DMT) drugs, together with contrast agents (gadolinium-diethylene triamine pentaacetic acid-Magnevist^®^ and ^125^I), were covalently conjugated with IgG4.1, an anti-amyloid antibody targeting cerebrovascular amyloid deposits in Alzheimer’s disease. A remarkable brain vasculature distribution was demonstrated, as well as the potential to reduce cerebrovascular inflammation associated with amyloid angiopathy [45]. The same authors developed similar systems by conjugating a putrescine modified F(ab′)_2_ fragment of IgG4.1 on the surface of CH-Magnevist^®^ nanoparticles encapsulating the immunosuppressant cyclophosphamide (CYP). Both in vitro and in vivo studies demonstrated improved ability to target cerebrovascular amyloid and reduce pro-inflammatory cytokine production by the amyloid beta proteins within the BBB endothelium compared to free CYP (Figure 4) [46].

The antibody active targeting approach was also explored for an alternative HIV treatment. Transferrin antibody (TfR) and Bradykinin B2 antibody (B2R)-modified nanoparticles delivering small interfering RNA (siRNA) across the BBB were proposed as effective tools to target HIV-infected brain astrocytes and inhibit virus replication [47]. In more detail, TfR and B2R were chemically conjugated to CH nanoparticles prepared by a complex coacervation method in the presence of siRNA, obtaining a significant increase in gene silencing efficiency in astrocytes compared to non-modified or single-antibody-modified CH nanoparticles.

## 3. Stimuli-Responsive Targeting

In stimuli-responsive targeting (also known as tertiary targeting), the vectorization is triggered by physiological or pathological signals within the site of interest, which induce nanocarrier structural modifications and payload release [56]. The variation of pH, temperature and redox potential, together with the overproduction of reactive oxygen species (ROS), represent the most common endogenous signals, whereas the application of ultrasound, electric and magnetic fields is classified as exogenous stimuli [57,58,59,60,61]. Focalized ultrasound has been used in the clinic because of encouraging localized bio-effects in preclinical models, but is also emerging as a valuable strategy for increasing vascular permeability and improve the therapeutics delivery in a nondestructive manner [62]. Conversely, electrochemotherapy was proposed to disrupt the endothelial membrane and facilitate the uptake of chemotherapeutics by using sublethal pulsed electric fields [63].

In the literature, several examples of stimuli-responsive PSC are proposed for the treatment of different brain diseases and disorders, with a broad range of sizes and architectures, such as nanoparticles, micelles, and hybrid systems. Interestingly, many of these combine both the targeting effect of the ligand/receptor interaction and stimuli-responsiveness (Table 2).

A pH-responsive prodrug was obtained conjugating HA with DOX and Lactoferrin (Lf) by an acid-labile hydrazone linkage and a carbodiimide-mediated reaction, respectively [64]. Each prodrug component contributed to conferring high selectivity for the glioma environment. In detail, HA and Lf (ligands for CD44 and Lf receptors, respectively), facilitate transport across the BBB, while the presence of pH-sensitive hydrazone moieties, cleavable at acidic pH, enhance the drug release in the tumor microenvironment. In vivo studies showed enhanced accumulation of Lf-HA-DOX in the tumor tissue, as well as longer median survival of glioma-bearing mice compared to the untreated control group. 

In recent years, redox-sensitive nanocarriers have been designed to trigger burst release of the loaded drug in response to the redox potential across the cell membrane due to different concentration of Glutathione (GSH) tripeptide [74]. This condition is typical of many solid tumors including glioma [75]. Redox potential-dependent systems usually contain disulfide linkages that are stable in the blood circulation and in the extracellular space, where the GSH concentration is low (approximately 2–20 μM). Conversely, these are able to be rapidly reduced through thiol-disulfide exchange reactions in the cytoplasm of cancer cells, where GSH is present in millimolar concentrations (approximately 2–10 mM), inducing a carrier structural modification [76]. 

Micelle systems (named HSC) for redox-responsive release of CUR in glioma cells were obtained by conjugating the polyphenol via disulfide linkage to low, medium and high molecular weight HA [65]. The study highlighted the importance of the HA molecular weight on carriers’ performance, with only the low and medium molecular weight HA-based conjugates being sensitive to GSH and toxic towards glioma cells. In a subsequent work, the same authors used Tween-80 as active targeting agent in HSC micelles, obtaining hemocompatible materials with distinct plasma stability, enhanced brain penetration capacity, and significant cytotoxicity against glioma cells compared to plain CUR and not redox-responsive micelles [66].

The overproduction of ROS, a characteristic biological event of many pathological conditions such as the ischemic stroke, can be used as an endogenous signal triggering drug release from smart delivery systems [77].

**Figure 5:**
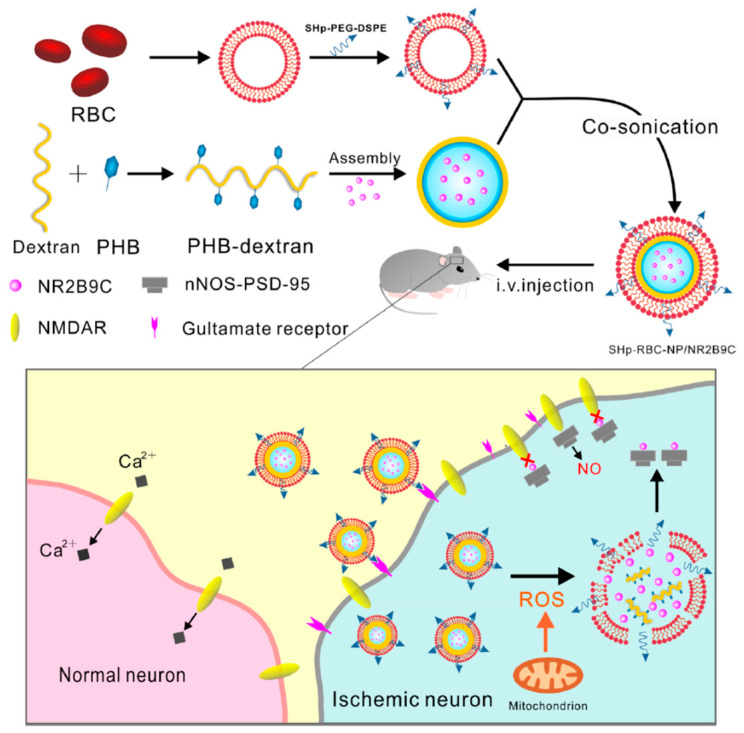
Schematic representation of SHp-RBC-NP/NR2B9C. Reproduced with permission from [67]. ACS (2018).

Bioengineered “core-shell” nanoparticles sensitive to ROS (coded as SHp-RBC-NP) were proposed for specific delivery of neuroprotective agent NR2B9C, prepared by modification of DEX with ROS-responsive boronic ester and a red blood cell (RBC) membrane with stroke homing peptide (SHp). In vitro studies demonstrated a protective effect on glutamate-induced cytotoxicity in PC-12 cells, while in vivo studies in models of middle cerebral artery occlusion in rat demonstrated a prolonged systemic circulation of NR2B9C, an enhanced targeting ability to ischemic tissues, and a reduced brain damage (Figure 5) [67].

Drug targeting induced by an external magnetic field can be achieved when a drug delivery vehicle possesses a strong magnetic moment [78,79,80]. In these systems, PS are used as coating materials of magnetic nanoparticles to improve the biocompatibility, penetration, and drug accumulation in the brain [68,81]. Hybrid superparamagnetic nanoparticles obtained by CH and DEX coating of magnetite (CH-DEX-SPIONs) were intravenously administered in orthotopic C6 gliomas in rats. This system showed high accumulation of the nanoparticles in the tumor site, as demonstrated by high-resolution magnetic resonance imaging studies [69]. Moreover, CH and Polyethyleneimine (PEI)-coated magnetic micelles (CPMMs) loading tomato-plasmid (ptd) were intranasally administered to rats after mild traumatic brain injury (mTBI) or sham surgery [70]. Main study outcomes included a remarkable increased accumulation of genic material in the brain, the absence of an inflammatory response, and the efficient excretion from the body. This demonstrates the suitability of CPMMs as a theranostic vehicle of genic material in mTBI.

The cellular uptake of magnetic nanoparticles was also improved by combining the stimuli-responsiveness to ligand-receptor internalization approaches. To this aim, in different works, Tf, Lf and chlorotoxin (CTX) were conjugated to magnetic nanoparticles coated with various polysaccharides.

Tf-conjugated CH-SPIONs were loaded with DOX and rhodamine B isothiocyanate (RBITC), in order to obtain a fluorescent theranostic vehicle against human brain tumors [71]. Such Tf-CH/SPIONs nanocarriers demonstrated an immediate response under magnetic field, stability in different media, efficient drug encapsulation, and cytocompatibility.

Magnetic nanoparticles coated with a redox-responsive chitosan-PEG copolymer and functionalized with O^6^-benzylguanine (BG) and chlorotoxin (CTX) were co-administered with Temozolomide (TMZ), a drug used for the treatment of GBM, to avoid TMZ inactivation and enhance its therapeutic performance [72]. A controlled and localized BG release under reductive intracellular conditions and the potentiation of TMZ effectiveness were observed in in vitro experiments. Moreover, in vivo assays, performed by simultaneously administering nanoparticles and TMZ, showed a 3-fold increase in median overall survival and a reduced myelosuppression compared to that observed with free BG when concurrently administered with TMZ.

**Figure 6:**
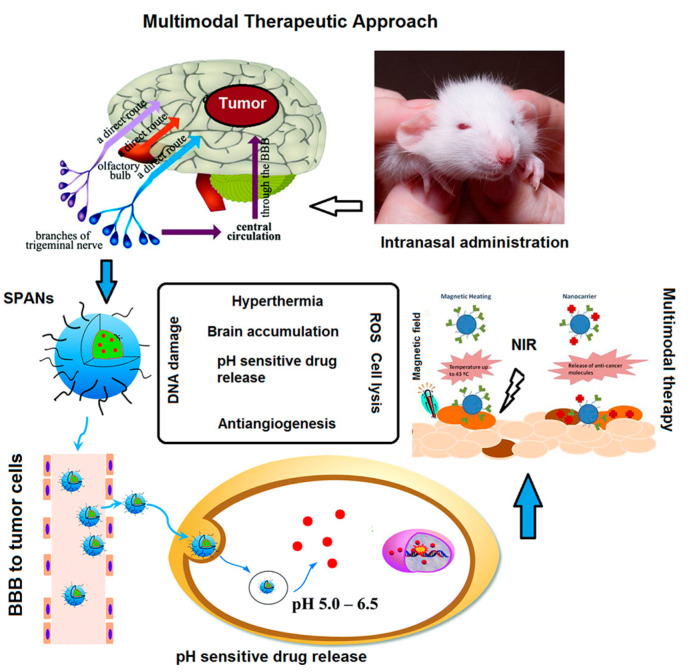
Effectiveness of hyaluronic acid-based pH-responsive alloy–drug nanoconjugates for glioblastoma treatment. Reproduced with permission from [73]. Elsevier (2019).

Finally, a multifunctional nanoconjugate, obtained by modification of FePt alloy nanoparticles with HA and Lf, was covalently bound to the drug lenalidomide (LND) through a pH-sensitive hydrazone bonding (Figure 6) [73].

This complex featured excellent heating ability upon exposure to alternating magnetic field and near-infrared laser irradiation, as well as with release of drug triggered by the acidic microenvironment of lysosome. Moreover, the leaching of Fe and Pt content determined a remarkable cytotoxic activity in U87 cells due to the increased ROS production, whereas the presence of Lf moieties, enhanced mucus penetration, allowing efficient brain uptake in in vivo experiments.

## 4. Targeting by Administration Route

Due to the peculiar structural and functional characteristics of the brain, the selection of the administration route is crucial to facilitate the drug accumulation and represents a real and effective drug targeting method.

Intracerebral devices represent the most direct tools for drug delivery to the target site and PSC scaffolds were used for the local treatment of brain tumors, Parkinson’s disease, and traumatic brain injury (Table 3).

A biodegradable fibrous scaffold composed of acetylated DEX (Ace-DEX) with tunable degradation rate was loaded with paclitaxel (PTX), and its performances tested as delivery device in a GBM in vivo model [82]. The study was performed on models of surgical resection and recurrence and distant metastasis, demonstrating that different survival outcomes can be obtained by changing the rate at which the same dose of drug is delivered. Ace-DEX scaffolds with a fast release rate were efficient in the distant metastasis treatment and, due to the pH sensitivity, allowed a selective PTX release in the tumor acidic environment.

Electro-responsive scaffolds based on alginate (ALG), aniline pentamer-functionalized CH, and agarose (AGAR) were loaded with a cocktail of neurotrophic factors and successfully employed as substrate for the differentiation of ecto-mesenchymal stem cells (OE-MSCs) into dopaminergic neuron-like cells [83]. Realtime PCR, immunocytochemistry, and flow cytometry confirmed the differentiation capacity of cells on conductive hydrogel, demonstrating the suitability of the proposed systems for the treatment of cerebral disorders, such as Parkinson’s disease.

HA-collagen scaffold was proposed as in vivo platform to deliver fibroblast growth factor (bFGF-CRS) and neural stem cells (NSCs) into the CA1 zone of the rat TBI area, promoting survival, neuronal differentiation of transplanted NSCs, and functional synapse formation, thus leading to the cognitive function recovery of the animals [84].

**Figure 7:**
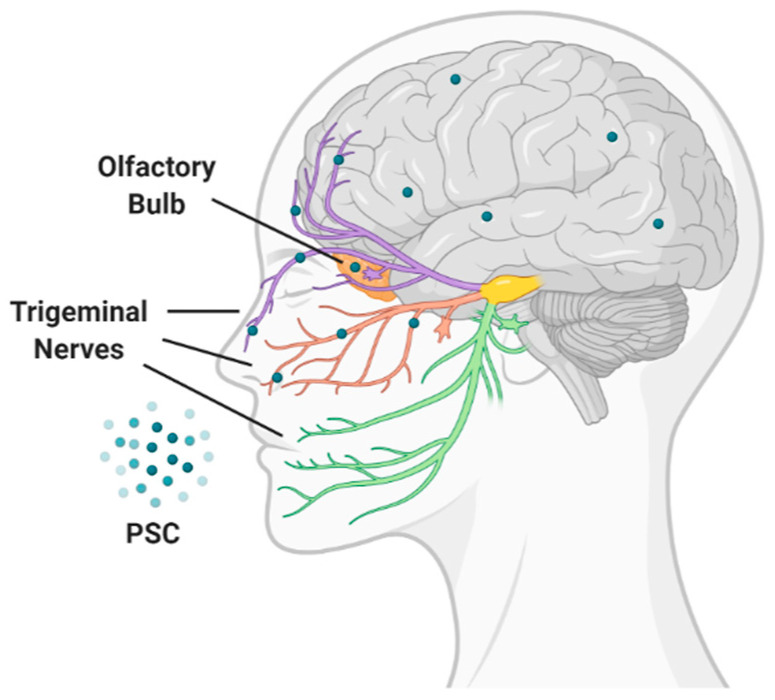
Nose to brain direct administration route of PSC.

The limitations of the invasive nature of intracranial and systemic administration routes can be overcome by the intranasal administration. This route allows the direct transport of drug-loaded nanoparticles from the nasal cavity to the brain through the olfactory and trigeminal nerves and cerebrospinal pathways, as well as the protection of the payload from mucosal enzyme damage (Figure 7) [118].

Due to its well-known mucoadhesive and penetration enhancement properties, CH is one of the most used excipients for nasal formulations [119], and several CH formulations have been developed for nose-to-brain (NB) drugs delivery [118]. Carboxylated CH–dopamine (DA) and CH–tyrosine (Tyr) conjugates were prepared to improve the brain delivery of the neurotransmitter DA. In vitro mucoadhesive assay as well as toxicity and uptake experiments in olfactory ensheathing cells (OECs), demonstrated the ability of the conjugate to release the neurotransmitter in simulated nasal fluid (SNF), absence of any cell toxicity and excellent internalization properties [85]. CH nanoparticles for NB delivery were prepared by different methods, such as desolvation, ionic crosslinking, emulsification or cold gelation.

Desolvation method was employed to prepare nanoparticles of Carboxymethyl chitosan (CMCH), a water-soluble derivative of CH, as an intranasal carrier of carbamazepine (CBZ), a drug used for the clinical treatment for seizure disorders, trigeminal neuralgia, and manic depressive illness, demonstrating enhanced drug bioavailability and brain targeting in in vivo experiments [86].

Donepezil (DNZ) [87] and rotigotine (RGT) [88] are a cholinesterase inhibitor and a DA agonist, respectively, whose use in the treatment of neurological disorders such as Alzheimer’s and Parkinson’s disease is limited by a poor pharmacokinetic profile and side-effects. Their formulation in CH nanoparticles obtained using tripolyphosphate (TPP) as polyanion crosslinker, enhanced brain targeting efficiency and drug bioavailability, as showed by in vivo experiments, confirming their potential as targeted delivery vehicles via olfactory nasal pathway.

Ionic gelation was also employed as crosslinking method for CH glutamate-based nanoparticles for the brain delivery of the anti-Parkinson drug rasagiline (RGN) [89]. Compared to the intravenous administration of both free drug and the drug-loaded nanoparticles, the intranasal administration of the formulation resulted in a significant enhancement of the brain drug concentration, and improved pharmacokinetic parameters. 

^99m^Technetium-labeled CH nanoparticles prepared via ionic gelation were employed for the nasal administration of zolmitriptan (ZMT), a serotonin receptor agonist used in migraine patients [90]. The amount of radioactivity (%) per gram of brain, the brain drug targeting, biodistribution, and brain kinetic parameters were investigated in mice and compared to that of intranasal pure drug solution and intravenous nanocarriers, demonstrating consistent better performance. The maximum concentration in the brain (C*_max_*) and the mean residence time (MRT) of intranasal administration increased by 1.57- and 1.27-fold vs. intranasal administration of the free drug, becoming 2.03 and 1.01 vs. intravenous administration of the nanocarrier. Similarly, the intranasal administration of MTX-loaded CH nanoparticles was found to be more effective in targeting central nervous system malignancies than the intravenous formulation of the same nanogel [91].

The enhanced cerebral drug bioavailability after intranasal administration was confirmed for the flavonol rutin (RUT) after encapsulation in CH nanoparticles [92]. Compared to the intranasal administration of free drug, the developed nanoparticles showed a significantly higher AUC_0–t_ in all three areas (brain, lungs and plasma), with a 178 folds higher brain bioavailability. Moreover, the authors reported improved neurobehavioral activity and reduced infarction volume in middle cerebral artery occlusion (MCAO) induced cerebral ischemic rat model.

Nanoparticles with enhanced loading efficiency for Scutellarin (SCU), a flavon used for the treatment ischemic cerebrovascular disease, were obtained combining the polysaccharide CH with hydroxypropyl-β-cyclodextrin (HP-β-CD) [93]. In vivo studies on C57BL mice showed higher concentrations of SCU in brain and plasma after intranasal and oral administration of the SCU nanoparticles compared to SCU solution. CH nanoparticles were also employed to deliver siRNA targeting galectin-1 (Gal-1), a galactose-binding lectin overexpressed in GBM and associated with tumor progression [94]. A successful intracellular delivery of anti-Gal-1 siRNA resulted in decreased expression of Gal-1 in both murine and human GBM cells and in over 50% Gal-1 reduction in tumor bearing mice.

Quetiapine fumarate (QF), a second-generation atypical antipsychotic drug with a short plasma half-life, was loaded on two CH formulations obtained by ionic gelation [95] and water titration [96] methods. In both cases, compared to drug solution, a significantly higher brain/blood ratio and 2–3.8-folds higher nasal bioavailability was registered.

CH intranasal emulsions for the brain delivery of rivastigmin (RIV), ZMT, and CUR were also designed. Ex-vivo experiments on CUR loaded nanoemulsions showed higher flux and permeation across sheep nasal mucosa compared to free CUR [97], whereas in vivo pharmacokinetic studies proved that upon intranasal administration, micro and nanoemulsions containing RIV [98] and ZMT [99] were effective in enhancing cerebral drugs concentration compared to other formulations administered intranasally or to intravenous/nasal solution of plain drugs.

Neuronanoemulsions for the treatment of Parkinson’s disease produced using a hot high-pressure homogenization technique were surface-modified with *N*,*N*,*N*-trimethyl chitosan (TMCH) to confer mucoadhesivity and loaded with high partitioning ROP-DEX sulfate nanoplex [100]. The formulation was found to be safe and stable over time, while in vivo studies exhibited high brain targeting efficiency through NB delivery via olfactory pathway. The same preparation technique was employed to develop TMZ nano lipid chitosan hydrogel formulations, showing a prolonged drug release, high permeation in nasal mucosa and significant TMZ concentration in brain, as demonstrated by in vivo studies [101].

Moreover, nanoparticles composed of PLGA and CH, obtained by solvent emulsion evaporation technique, were tested in NB delivery of desvenlafaxine (DVX), an antidepressant drug [102]. The system, intranasally administered in rats, determined a significant reduction of the depression symptoms together with increased levels of monoamines in the brain in comparison with the orally administered drug.

CH-aspartate (CH-Asp) and HP-β-CD incorporated in oil in water microemulsions were proposed as NB delivery vehicle of buspirone hydrochloride (BUS). A remarkable improvement of the area under the curve (AUC) and the targeting efficiency in comparison with the intravenous administration was demonstrated by in vivo pharmacokinetic studies [103].

CH-coated PLGA and PCL nanoparticles for intranasal Rasagiline (RGN) [104], cathechin hydrate (CAT) [105] and glycyrrhizic acid (GA) [106] release were proposed for the treatment of various brain disorders. In all studies, pharmacokinetic experiments conducted in Wistar rats showed a significant high bioavailability and C_max_ over the intravenous treatment group, confirming the suitability of nanoparticulate system as targeted brain dosage forms of hydrophilic molecules with undesirable side effects and high-hepatic first-pass metabolism.

Lipid microparticles uncoated or coated with CH were loaded with the neuroprotective polyphenol resveratrol (REV) and administered via intranasal route [107]. In vivo studies demonstrated a dramatic increase in the levels of REV reaching the cerebrospinal fluid after intranasal administration of an aqueous suspension of REV-loaded nanoparticles, compared to free drug or uncoated nanoparticles, without any distribution in the systemic circulation.

In another work, ROP-DEX sulphate (ROPI-DS) nanoplex was encapsulated in nanostructured-lipid carriers surface-modified with mucoadhesive TMCH [107]. The system was evaluated for direct NB delivery (via olfactory and/or trigeminal pathway) in a mice model, finding high drug concentrations in the brain. Surface-modified lipid nanoparticles were also employed as carriers of glial cell-derived neurotrophic factor (GDNF), a widely studied growth factor used in Parkinson’s disease [108]. The nanoparticles were coated with a CH derivative obtained by covalent conjugation with a transactivator of transcription (TAT) peptide, acting as cell-penetrating agent, to increase drug permeating efficiency and tissue targeting. Intranasal administration of this system in a mouse model of Parkinson’s disease resulted in motor recovery and a significant reduction of microgliosis. Finally, aerosol formulations of anionic liposomes coated with *N*-(2-hydroxy)propyl-3-trimethyl ammonium CH chloride were loaded with Ghrelin (GHRL) and proposed as potential tool for cachexia treatment, showing an enhanced GHRL permeation through the Calu3 epithelial monolayer and increased bioadhesion [109].

Due to the physical characteristics of the application site, the ideal drug delivery formulation for NB administration should possess specific features: liquid forms are poorly retained by the nasal mucosa due to quick clearance from nasal cavity [120], whereas the application of nasal gel [121,122], although demonstrating a longer retention time, is hindered by difficulties in achieving accurate dosing and poor patients compliance. In situ thermoresponsive gels satisfy all the requirements of an ideal nasal formulation, being liquids at room temperature and in a semi-solid state with mucoadhesive behavior at physiological temperature.

Polymers such as pluronic F-127 and pluronic F-68, due to their advantageous features in terms of biocompatibility and good release profile, were widely used in thermosensitive gelling systems for intranasal and intraocular drug delivery [123,124]. However, some drawbacks related to reduced drug absorption due to low residence time were recorded, necessitating the association with mucoadhesive polymers, such as CH, to maximize drug absorption.

Thermosensitive in situ gels composed of a mixture of pluronic F-127, pluronic F-68, and CH were proposed for the treatment of HIV-associated neurological disorders [110] and polyglutamine diseases [111], a set of progressive neurodegenerative disorders caused by misfolding and aggregation of mutant CAG RNA and Polyglutamine proteins. In both studies, in vivo experiments showed a marked increase of the brain uptake compared to the free drug solution.

Moreover, the insertion of polyaniline in CH, HPMC, and pluronic F127 blend added electro-responsivity properties to the resulting thermoresponsive gel, allowing the drug release profile to be modulated by an external electric field for a controlled NB delivery [112].

Besides CH, other polysaccharides such as HA, gellan gum (GLG), xanthan gum (XG), and tamarind seed polysaccharide were employed as base material of NB formulations. Mucoadhesive core-shell nanoparticles, composed by organic or inorganic cores surrounded by HA corona, were proposed to deliver drugs or gene materials, to limit the mucociliary clearance and maximize the therapeutic potential of the cargoes. In this case, an amphiphilic derivative of octaarginine was complexed with miRNA and enveloped with different protective polymers, (e.g., polyethyleneglycol, polyglutamic acid or HA), as enhancers of stability and mucodiffusion across the olfactory nasal mucosa, with a significant increase of miRNA mimic release in the hippocampus area in a mouse model of Alzheimer’s disease [113].

A diethylaminoethyl-dextran (DAEDEX)/HA mixture was employed as shell of mesoporous Calcium carbonate particles loaded with the anxiolitic drug zolpidem (ZPD) [114]. When intranasally administrated in mice, the mucoadhesive coating improved drug bioavailability resulting in a pronounced anxiolytic effect on the animal behavior.

A water solution of HA was used as dispersing phase of a lipidic nanoemulsion for the co-administration of REV and CUR [115], polyphenols with potential therapeutic application in neurodegenerative diseases. In vitro and ex vivo release experiments of the two polyphenols, the evaluation of safety on nasal mucosa, and in vivo quantification of the two drugs in rat brains were performed, demonstrating that when loaded in the HA-based nanoemulsion, the polyphenols were protected against degradation and released in a controlled manner, resulting in brain concentration 7–9-folds higher than that recorded administrating the free drugs solution.

REV was also loaded in lipid carrier and nanostructured in GLG and XG [116]. The resulting in situ gel was formulated and characterized, obtaining a fivefold higher permeation across the nasal mucosa compared to REV suspension-based in situ gel. Moreover, in vivo pharmacodynamic study in the scopolamine-induced amnesia rat model, demonstrated a significant improvement in memory function. In situ gel composed of deacetylated GG was also formulated by ion activation mechanism in simulated nasal fluid for nasal delivery of sumatriptan succinate (SMP), a 5-hydroxy tryptamine receptor agonist used in the treatment of migraine and cluster headache and hampered by poor bioavailability and toxic side effects when orally administered [117]. Animal studies showed higher AUC in plasma and brain after intranasal gel delivery compared to oral administration. Moreover, higher AUC value in brain was demonstrated after intranasal application, compared to plasma.

## 5. Conclusions and Perspectives

In recent years, high expectations have been placed on the development of drug delivery systems as smart strategies to circumvent the BBB, enhancing the drug vectorization to the brain. Due to their chemical versatility and advantageous biological features, PSC nanocarriers are explored as valuable tools to cross the BBB, primarily designed using the main targeting mechanisms, namely passive and active transport, administration route, and stimuli-responsivity. 

The reviewed studies cover a broad number of pathologies, and the design of the most suitable vehicle, in terms of choice of PSC, morphology, and (possible) chemical derivatization, strongly depends on the disease features.

Thus, in order to give an exhaustive overview of the state of the art in the field, the results of the studies discussed in this paper are summarized in Table 4. 

The reviewed studies were initially classified according to the pathology. Next, for each brain disease, the incidence (% of reviewed studies) in the use of the different polysaccharides for the nanocarrier synthesis was calculated, together with the percentage of the applied administration route, vectorization strategy, and obtained efficacy (in vitro vs. in vivo assays).

It is evident that most nanoparticles were locally administrated, with the NB resulting the widely applied route, as per data discussed in Table 3, due to both anatomic factors (the direct communication between the nasal and the brain structures) and the muco-adhesion of many PSC materials.

When the systemic administration is explored, mostly for the treatment of brain tumors and Alzheimer’s disease, the proposed materials possess as common feature the prolonged circulation times, resulting in an enhanced BBB permeation ability and high drug bioavailability in the brain.

Passively targeted nanoparticles, mainly applied in the treatment of neurodegenerative diseases, are prepared from CH and its derivatives, because of its positive charge and ability to temporarily open the tight junction of the BBB endothelial cells.

In addition, CH muco-adhesion is widely exploited for the development of effective NB formulations for the treatment different pathologies, such as psychiatric and neurological disorders, migraine, ischemic stroke and HIV-associated neurocognitive disorders.

For their localization and histopathological characteristics, the treatment of brain tumors is particularly difficult, and PSC nanoparticles employed for this purpose are vectorized using both active targeting, by covalent linkage of specific ligands, and the stimuli-responsivity to endogenous or exogenous signals. In addition, most of the works reported on the use of HA, as base material of nanoparticles in brain tumors treatment is due to its affinity for CD44 receptors, overexpressed on glioma and GBM cells, thus conferring to the final material an intrinsic targeting ability.

Overall, because the majority of studies are well supported by in vivo assays, the bench to clinic translation of PSC vehicles should not be a chimera, however some considerations about their effective contribute in facing the challenge of drug targeting to the brain should be done. First of all, natural polysaccharides, being very heterogeneous in terms of molecular weight and composition, are characterized by variable physicochemical properties (i.e., solubility, chain flexibility, intra- and intermolecular forces, and surface charge) [125]. Since these features strongly influence the behavior of PSC when applied in vivo, a regulatory validation of each manufacturing step is needed in the view of a clinical application. Beside performance and functionality, the scale-up and reproducibility, the process repeatability and reliability, the production efficiency and yield, as well as the toxicity, environmental, health and safety requirements are the critical issues to be considered [126].

For the preparation of brain-targeted PSC, ionic gelation and emulsification are the most feasible techniques to be translated to a large-scale production due to the low number of parameters to be set [127].

Furthermore, often the available in vitro and in vivo models do not fully mimic the human physiology and physiopathology; thus, a critical evaluation of the preclinical data is required before setting-up clinical trials [128].

In conclusion, the clinical translation of PCS requires a deep integration of academia and industrial expertise, in a multidisciplinary exchange of knowledge involving chemists, physicians, material scientists, engineers, biologists, clinicians, with the further contribution of regulatory authorities.

## Figures and Tables

**Table 1:** Polysaccharide carriers for the treatment of brain diseases by passive and active targeting.

PS Ref	System (Preparation)	Targeting	AR	Disease *Drug*	Performance		
					In Vitro	In Vivo	Outcome
CH [21]	NP (γ-ray)	Enhanced permeation	Oral	Alzheimer *MEM*	---	Albino rats	Neurological amelioration * #
AGCH [22]	NP (ion gelation)	Enhanced permeation (AG moieties)	systemic	--- ---	ECs bEnd3	---	Enhanced uptake ^§^
AGDEX [23]	NP (e-polym)	Enhanced permeation (AG moieties)	systemic	--- *DOX* *CUR*	bEnd3	---	Sustained release ^§^
AGDEX [24]	NP (el-spray)	Enhanced permeation (AG moieties)	systemic	--- *DOX*	bEnd3	---	Enhanced uptake ^§^
AGDEX [25]	NPs (e-polym)	Enhanced permeation (AG moieties)	systemic	--- *Ang II* *AnoxPep*	---	Trout/ mice	Neurological amelioration *
AGPS [26]	NP (chem-cross)	Enhanced permeation (AG moieties)	systemic	-- *DOX;* *Ang II*	bEnd3	---	Enhanced uptake ^§^
HA/CH [27]	CSNP (e-polym)	Enhanced permeation (CH)	systemic	Ischemia *NGB*	---	Wistar rat	Neurological amelioration #
PGCH [28]	CNF (sonication)	Enhanced permeation (PG moieties)	systemic	--- *ENK-pp*	---	CD-1 mice	Improved pharmacokinetics #
HA [29]	COLL-HA (complex)	Prolonged delivery	topical	Parkinson *Tat-Hsp70*	SH-SY5Y	CD-1; C57Bl/6N mice	Neurological amelioration * #
MDX [30]	NP (chem-cross)	Enhanced permeation	systemic	Parkinson *TH*	SH-SY5Y	Wild-type Bl-6 mice	Neurological amelioration #
CMC [31]	NPQD (magnetic stir)	Enhanced permeation	systemic	GBM *DOX*	U87	---	Enhanced uptake ^§^
CMC [32]	NPQD (magnetic stir)	Enhanced permeation	systemic	GBM ---	U87	---	Enhanced uptake ^§^
CH [33]	NP (ion gelation)	Ligand-receptor (Tween 80)	systemic	--- ---	---	SD rats	Neurotoxicity #
CH [34]	NP (ion gelation)	Ligand-receptor (Tween 80)	systemic	Migraine *NYS*	---	Swiss albino mice	Neurological amelioration *
CH [35]	NP (em-cross)	Ligand-receptor (Tween 80)	systemic	Parkinson *ROP*	---	Wistar rats	Improved pharmacokinetics #
CMC [36]	CSNP (self-assembly)	Ligand-receptor/ transporter (RGD/L-Arg)	---	GBM *DOX*	U-87	CAM	Enhanced uptake ^§^ Synergism #
CH/HA [37]	NP (mixing)	HA	systemic	Glioma *CUR*	C6	---	Enhanced uptake ^§^
CH/HA [38]	NP (mixing)	HA	systemic	Glioma *CUR*	C6	SD rats	Enhanced uptake ^§^ Improved pharmacokinetic #
HA [39]	LPsNP (film hydration)	HA	systemic	Glioma *DOX*	C6	BALB/c mice-SD rats	Enhanced uptake Synergism #
HA [40]	LNP (film hydration)	HA	---	GBM *DOX*	A172; U87MG	---	Enhanced uptake ^§^
HA [41]	NPs (solvent evap)	Pep	systemic	GBM *DTX*	C6; U87; U251; BMVEC; NIH/3T3	SD rats; BALB/c mice	Enhanced uptake ^§^ Synergism #
HP [42]	NPs (self-assembly)	cRGD; SWLAYPGAVSYR Peptides	systemic	Glioma ---	U251; U87; HUVEC; HEB	U251-xenograft mice	Enhanced uptake ^§^ Improved pharmacokinetics #
CH [43]	NP (ion gelation)	L-Val	systemic	Alzheimer *SGT*	---	Wistar rats	Improved pharmacokinetics #
HA [44]	Prodrug (grafting)	NAA	---	Brain tumor	---	---	Molecular docking ^§^
CH [45]	NP (ion gelation)	Magnevist^®^/ IgG4.1	systemic	Alzheimer *CUR/DMT*	hCMEC;D3	B6/SJL mice; Tg2576 transgenic mice	Improved pharmacokinetics #
CH [46]	NP (ion gelation)	Magnevist^®^/ F(ab′)_2_	systemic	CAA *CYP*	hCMEC;D3	B6SJLF1/J mice	Improved pharmacokinetics #
CH [47]	NP (ion gelation)	TfR/B2R	systemic	HIV *SiRNA*	U138-MG; hCMEC;D3	---	Enhanced uptake ^§^

^§^ From in vitro data; * in vivo by animal behavior performance; # in vivo by analytical assessment; AGCH: alkylglyceril chitosan; AGDEX: alkylglyceril dextran; AGPS: alkylglyceryl-modified polysaccharides; Ang II: angiotensin II; AnoxPep: anorexigenic octaneuropeptide; AR: administration route; B2R: bradykinin B2 antibody; CAA: cerebral amyloid angiopathy; CAM: chorioallantoic membrane; CH: chitosan; Chem-cross: chemical crosslinking; CMC: carboxymethylcellulose; CNF: coated nanofibers; CSNP: core-shell nanoparticles; CUR: curcumin; CYP: cyclophosphamide; DMT: dexamethasone; DOX: doxorubicin; DTX: docetaxel; ENK-pp: enkephalin-peptide prodrugs; El-spray: electrospray; Em-cross: emulsification crosslinking; Em-polym: emulsion polymerization; Evap: evaporation; GBM: glioblastoma; HA: hyaluronic acid; HP: heparin; l-Arg: l-arginine; LNP: lipid nanoparticles; LPsNP: liposomes coated nanoparticles; l-Val: l-valine; MDX: maltodextrin; MEM: memantine; NAA: neutral amino acids; NGB: oxygen-binding neuroglobin protein; NH: nanohybrids; NPQD: nanoparticles quantum dots; NYS: nystatin; O-FCD: oligo-fucoidan; Pep: functional peptide; PGCH: *N*-palmitoyl-*N*-monomethyl-*N*,*N*-dimethyl-*N*,*N*,*N*-trimethyl-6-*O*-glycolchitosan; PS: polysaccharide; RGD: integrin target receptor tripeptide; ROP: ropinirole hydrochloride; SD rats: Sprague-Dawley rats; SGT: saxagliptin; TBI: traumatic brain injury; TfR: transferrin antibody; TH: tyrosine hydroxylase.

**Table 2:** Stimuli-responsive PSC in brain diseases.

PS Ref	System (Preparation)	Stimuli	Active Targeting	AR	Disease *Drug*	Performance		
						In Vitro	In Vivo	Outcome
HA [64]	Prodrug (condensation)	pH	Lf	systemic	Glioma *DOX*	U87 C6	C6 orthotopic mice	Enhanced uptake ^§^ Improved pharmacokinetics # Synergism #
HA [65]	Micelles (self-assembly)	Redox	---	systemic	Glioma *CUR*	G422	---	Enhanced uptake ^§^
HA [66]	Micelles (self-assembly)	Redox	Tween80	systemic	Glioma *CUR*	BEnd3 G422	---	Enhanced uptake ^§^
PHB-DEX [67]	NP (sonication)	ROS	---	systemic	Stroke ---	PC-12; BCECs	SD rat	Improved pharmacokinetics # Neurological amelioration #
CMC [68]	NP (precipitation)	Magnetic	---	---	--- *DPM*	HLMVE	---	Enhanced uptake ^§^
CH/ DEX [69]	NP (ion gelation)	Magnetic	---	systemic	GBM ---	C6; U87	C6 xenograft	Enhanced uptake ^§^ Synergism #
CH [70]	micelles (solvent evap)	Magnetic	---	NB	TBI *DNA*	HT22	SD rats	Enhanced uptake § Improved pharmacokinetics #
CH [71]	NP (precipitation)	Magnetic	Tf	systemic	GBM *DOX*	U251 MG	---	Enhanced uptake §
CH [72]	NP (precipitation)	Magnetic/ Redox	CTX	CED	GBM *BG*	SF767	GBM6 mice xenograft	Enhanced uptake § Improved pharmacokinetics # Synergism #
HA [73]	NP (chem-coat)	Magnetic/ pH	cAA; Lf; HA	NB	GBM *LND*	U87MG	Wistar rats	Enhanced uptake § Improved pharmacokinetics #

^§^ From in vitro data; * in vivo by animal behavior performance; # in vivo by analytical assessment; BG: O6-benzylguanine; cAA: cis-aconitic anhydride; CED: convection-enhanced delivery; CMC: carboxymethylcellulose; CTX: chlorotoxin; CUR: curcumin; DEX: dextran; DOX: doxorubicin; DPM: dopamine; GNM: glioblastoma; HA: hyaluronic acid; Lf: lactoferrin; LND: lenalidomide; NB: noise to brain; PHB-DEX: boronic acid conjugated dextran; ROS: reactive oxygen species; SD: Sprague-Dawley rats; TBI: traumatic brain injury; Tf: transferrin.

**Table 3:** Brain targeting strategies modifying the PSC administration route.

PS Ref	System (Preparation)	AR	Disease *Drug*	Performance		
				In Vitro	In Vivo	Outcome
Ace-DEX [82]	NF (extrusion)	Local	GBM *PTX*	U87	Athymic nude mice	Controlled release ^§^ Improved pharmacokinetics #
CH/ALG/ AGAR [83]	Hydrogel (cryogelation)	Local	Parkinson *DMT*	OE-MSCs	---	Neuronal differentiation ^§^
HA [84]	Scaffold (mixing)	Local	TBI *bFGF*	NSCs	TBI rats	Stem cell differentiation ^§^ Neurological amelioration #
CCH [85]	Conjugate (chem-coup)	NB	Parkinson *DA* *Tyr*	OECs	---	Enhanced uptake ^§^
CMCH [86]	NP (desolvation)	NB	Epilepsy *CBZ*	---	C57BL mice	Improved pharmacokinetics #
CH [87]	NSP (ion gelation)	NB	Alzheimer *DNZ*	---	SD rats	Improved pharmacokinetics #
CH [88]	NP (ion gelation)	NB	Parkinson *RGT*	SH-SY5Y	SD rats	Enhanced uptake ^§^ Improved pharmacokinetics # Neurological amelioration *#
CG [89]	NP (ion gelation)	NB	Parkinson *RGN*	GNM	Swiss albino mice	Enhanced permeation ^§^ Improved pharmacokinetics #
CH [90]	NP (ion gelation)	NB	Migraine *ZMT*	---	Swiss albino mice	Improved pharmacokinetics #
CH [91]	NP (ion gelation)	NB	CNS Lymphoma *MTX*	---	SD rats	Improved pharmacokinetics #
CH [92]	NP (ion gelation)	NB	Ischemia *RUT*	GNM	Wistar rats	Improved pharmacokinetics # Neurological amelioration #
CH [93]	NP (ion gelation)	NB	Ischemia *SCU*	---	C57BL mice	Improved pharmacokinetics #
CH [94]	NP (ion gelation)	NB	GBM *anti-Gal-1 siRNA*	GL261; HPC	GL261-WT; GL261-BFP orthotopic mice	Enhanced uptake ^§^ Neurological amelioration #
CH [95]	NP (ion gelation)	NB	Schizophrenia *QF*	GNM	SD rats	Enhanced permeation ^§^ Improved pharmacokinetics #
CH [96]	ME (water trit)	NB	Schizophrenia *QF*	GNM	SD rats	Enhanced permeation ^§^ Improved pharmacokinetics #
CH [97]	NE (emulsion)	NB	BBB overcoming *CUR*	SNM	---	Enhanced permeation ^§^
CH [98]	ME (water trit)	NB	Dementia *RIV*	SNM	SD rats	Enhanced permeation ^§^ Improved pharmacokinetics #
CH [99]	NE (water trit)	NB	Migraine *ZMT*	SNM	SD rats	Enhanced permeation ^§^ Improved pharmacokinetics #
TMCH [100]	NE (HP-homogen)	NB	Parkinson *ROP*	---	Swiss albino mice	Improved pharmacokinetics #
CH [101]	CSLNP (HP-homogen)	NB	Glioma *TMZ*	---	Winstar rats	Improved pharmacokinetics # Reduced toxicity #
CH [102]	CSNP (solv em evap)	NB	Depression *DVX*	porcine mucin	Wistar rats	Improved pharmacokinetics # Neurological amelioration * #
CH-Asp [103]	ME (water trit)	NB	Anxiety *BUS*	SNM	Wistar albino rats	Improved pharmacokinetics #
CH [104]	CSNP (solv em evap)	NB	Parkinson *RGN*	GNM	Wistar rat	Enhanced permeation ^§^ Improved pharmacokinetics #
CH [105]	CSNP (double em)	NB	Epilepsy *CAT*	GNM	Wistar rats	Enhanced permeation ^§^ Improved pharmacokinetics #
CH [106]	CSNP (solv em evap)	NB	Cerebral Ischemia *GA*	GNM	Albino Wistar Rats	Mucoadhesion ^§^ Enhanced permeation ^§^ Improved pharmacokinetics #
CH [107]	CSLM (melt em)	NB	--- *REV*	NCM460	Wistar rats	Enhanced permeation ^§^ Improved pharmacokinetics #
CH [108]	CSNP (melt em)	NB	Parkinson *GDNF*	---	C57BL/6J mice	Neurological amelioration #
CH [109]	CSL (film hydration)	NB	Cachexia *GHRL*	Calu3	---	Mucoadhesion ^§^ Enhanced permeation ^§^
CH [110]	Gel (cold method)	NB	HIV *DB213*	---	SD rats; C57BL/6J mice	Improved pharmacokinetics #
CH [111]	Gel (cold method)	NB	Polyglutamine diseases *QBP1/L1P3V8*	---	SD rats; C57 WT mice; R6/2 HD transgenic mice	Improved pharmacokinetics # Neurological amelioration * #
CH/ HPMC [112]	Gel (cold method)	NB	--- *CRM*	---	---	Controlled release ^§^
HA [113]	MP (ion interaction)	NB	Alzheimer ---	CHO	App^NL−G-F^ knock-in mouse	Enhanced uptake ^§^ Improved pharmacokinetics #
HA / DAEDEX [114]	CSNP (physical coating)	NB	Anxiolytics *ZPD*	---	BALB/c mice	Neurological amelioration *
HA [115]	NE (spontaneous em)	NB	Neurodegenerative diseases *REV; CUR*	SNM	Albino rats	Enhanced permeation ^§^ Improved pharmacokinetics #
GG/XG [116]	Gel (melt em-probe sonication)	NB	Alzheimer *REV*	SNM	SD rats	Enhanced permeation ^§^ Improved pharmacokinetics #
GG [117]	Gel (cold gelation)	NB	Migraine *SMP*	SNM	SD rats	Enhanced permeation ^§^ Improved pharmacokinetics # Neurological amelioration *

§ from in vitro data; * in vivo by animal behavior performance; # in vivo by analytical assessment; Ace-DEX: acetalated dextran; AGR: agarose; ALG: alginate; AR: administration route; BUS: buspirone hydrochloride; CAT: catechin hydrate; CBZ: carbamazepine; CCH: carboxylated chitosan; CH: chitosan; CH-Asp: chitosan aspartate; chem-coup: chemical coupling; CRM: carmustine; CSL: core-shell liposomes; CSLM: core-shell lipid microparticles; CSNP: core-shell nanoparticles; CUR: curcumin; DAE-DEX: diethylaminoethyl-dextran; DEX: dextran; DMT: dexamethasone; DNZ: donepezil; DVX: desvenlafaxine; em: emulsion; GA: glycyrrhizic acid; GBM: glioblastoma; GDNF: glial cell-derived neurotrophic factor; GHRL: ghrelin; GLG: Gellan gum; GNM: goat nasal mucosa; HPG: human primary glioblastoma cells; HP-homogen: high-pressure homogenization; ME: microemulsion; MTX: methotrexate; MP: microparticles; NE: nanoemulsion; NF: nanofibers; NPS: nanosuspension; PTX: paclitaxel; QBP1: polyQ binding peptide 1; QF: quetiapine fumarate; REV: resveratrol; RGN: rasagiline; RGT: rotigotine; RIV: rivastigmine; RLH: rat liver homogenate; RNA-NC: RNA nanocomplex; ROP: ropinirole hydrochloride; RUT: rutin; SCU: scutellarin; SLM: solid-lipid microparticles; SMP: sumatriptan succinate; SNM: sheep nasal mucosa; solv em evap: solvent emulsion evaporation; TAT: transactivator of transcription; TBI: traumatic brain injury; TMCH: trimethylchitosan; trit: tritation; TMZ: temozolomide; XG: xanthan gum (XG), ZDV: zidovudin; ZMT: zolmitriptan; ZPD: zolpidem.

**Table 4:** Outcomes of PSC proposed for the treatment of brain diseases reviewed in this paper. Percentages are based on the total studies of each disease route.

Disease (Ref)	PS	Load	AR		Vectorization				Efficacy	
			S	L	Adm	Stimuli	Passive	Active	In Vitro	In Vivo
Alzheimer [21,43,45,46,87,98,113,115,116]	CH (70) HA (20) GG (10)	60	40	40	40	--	10	40	50	80
Parkinson [29,30,85,88,89,100,104,108,115]	CH (67) HA (17) MDX (8)	83	33	50	58	--	25	17	58	83
Brain tumors [31,32,36,39,40,41,42,44,64,65,66,69,71,72,73,82,91,94]	HA (47) CH (24) CMC (12) HP (6) DEX (6)	74	58	21	16	37	16	58	74	42
Ischemia [27,67,86,92,106]	CH (60) HA (20) DEX (20)	80	40	60	60	20	20	--	60	100
TBI [70,84]	CH (50) HA (50)	50	50	50	50	50	50	--	50	100
Migraine [34,90,99,117]	CH (75) GG (25)	100	25	75	75	--	25	--	50	100
HIV [47,110,119]	CH (100)	100	33	67	67	0	0	33	67	67
Psychiatric disorders [95,96,102,103,105,114]	CH (80) HA (20)	100	--	100	100	--	--	--	80	100
Epilepsy [86,105]	CH (100)	100	--	100	--	--	100	--	50	100
Other [109,111]	CH (100)	100	--	100	100	--	--	--	50	50

Adm: administration; AR: administration route; CH: chitosan; CMC: carboxymethyl cellulose; DEX: dextran; GG: guar gum; HA: hyaluronic acid; HP: heparin; L: local; MDX: maltodextrin; S: systemic; TBI: traumatic brain injury.
